# The control of male fertility by spermatid-specific factors: searching for contraceptive targets from spermatozoon's head to tail

**DOI:** 10.1038/cddis.2016.344

**Published:** 2016-11-10

**Authors:** Su-Ren Chen, Aalia Batool, Yu-Qian Wang, Xiao-Xia Hao, Chawn-Shang Chang, C Yan Cheng, Yi-Xun Liu

**Affiliations:** 1 State Key Laboratory of Stem Cell and Reproductive Biology, Institute of Zoology, Chinese Academy of Sciences, Beijing, China; 2 University of the Chinese Academy of Sciences, Beijing, China; 3 George Whipple Lab for Cancer Research, University of Rochester Medical Center, Rochester, MN, USA; 4 The Mary M. Wohlford Laboratory for Male Contraceptive Research, Population Council, New York, NY, USA

## Abstract

Male infertility due to abnormal spermatozoa has been reported in both animals and humans, but its pathogenic causes, including genetic abnormalities, remain largely unknown. On the other hand, contraceptive options for men are limited, and a specific, reversible and safe method of male contraception has been a long-standing quest in medicine. Some progress has recently been made in exploring the effects of spermatid-specifical genetic factors in controlling male fertility. A comprehensive search of PubMed for articles and reviews published in English before July 2016 was carried out using the search terms ‘spermiogenesis failure', ‘globozoospermia', ‘spermatid-specific', ‘acrosome', ‘infertile', ‘manchette', ‘sperm connecting piece', ‘sperm annulus', ‘sperm ADAMs', ‘flagellar abnormalities', ‘sperm motility loss', ‘sperm ion exchanger' and ‘contraceptive targets'. Importantly, we have opted to focus on articles regarding spermatid-specific factors. Genetic studies to define the structure and physiology of sperm have shown that spermatozoa appear to be one of the most promising contraceptive targets. Here we summarize how these spermatid-specific factors regulate spermiogenesis and categorize them according to their localization and function from spermatid head to tail (e.g., acrosome, manchette, head-tail conjunction, annulus, principal piece of tail). In addition, we emphatically introduce small-molecule contraceptives, such as BRDT and PPP3CC/PPP3R2, which are currently being developed to target spermatogenic-specific proteins. We suggest that blocking the differentiation of haploid germ cells, which rarely affects early spermatogenic cell types and the testicular microenvironment, is a better choice than spermatogenic-specific proteins. The studies described here provide valuable information regarding the genetic and molecular defects causing male mouse infertility to improve our understanding of the importance of spermatid-specific factors in controlling fertility. Although a male contraceptive ‘pill' is still many years away, research into the production of new small-molecule contraceptives targeting spermatid-specific proteins is the right avenue.

## Facts

1. While some nations are experiencing a population explosion, others show negative growth. The future population growth rate is highly dependent on improving fertility *versus* contraception.
2. Male factor infertility is a complex disorder that affects a large sector of the population; however, most its etiology and genetic testes remain largely unexplored.
3. Few approaches other than barrier methods have been adequately developed for male contraception. Male hormonal contraception, which disrupts the entire spermatogenesis process, is not a preferred approach and will be discarded in the furture.
4. Most of the spermiogenic genes are highly conserved between mice and humans. Thus gene ablation in mice has been a powerful tool for identifying spermatid-specific proteins essential for spermiogenesis, which might serve as potential targets of male contraception.

## Open questions

1. What are the spermatid-specific factors required for spermiogenesis and how do they control male fertility in mice? Can they be classified into different groups by their localization and function in spermatids?
2. What do these knockout mice tell us? Which factors are likely to turn out to be the spermatid-specific targets for male contraceptives? What is the mechanism of contraceptive action, and does the ‘pill' target spermiogenic process specifically and reversibly?
3. Many genes have been shown to be associated with spermiogenesis in mice using knockout mouse models. However, the majority of mouse model studies fail to identify a mutation in infertile human males. Thus are the rodent models physiologically relevant to humans?

As many as 15% of human couples are infertile, and male infertility is associated with about half of these cases.^1^ The inability to procreate is frequently considered a personal tragedy and a curse for the couple, impacting on the entire family and even the local community.^2^ Currently, the pathophysiological mechanisms of male infertility are so poorly understood that most infertile men receive a description of ‘idiopathic oligo/asthenozoospermia' rather than a diagnosis; hence, specific medical treatment is not possible.^3^ Hormonal therapy has an important but limited role as an effective treatment of gonadotrophin deficiency but no established role in empirical therapy.^3^ Although assisted reproductive techniques (ART), such as *in vitro* fertilization (IVF) and intracytoplasmic sperm injection (ICSI), allow men with suboptimal sperm quality to overcome natural selection mechanisms and produce a viable zygote, the advent of ART has emphasized the necessity and importance of elucidating the genetic basis of male infertility because inheritance of mutations passed on through ART can cause unacceptable serious consequences.^4, 5, 6^ Given that many non-obstructive causes of male infertility are unexplained and the therapeutic effect is beyond the power of hormone and ART, focussing on genetic causes and identifying genes and pathways associated with infertility becomes a public health priority.^7^ On the other hand, few approaches other than barrier methods have been adequately developed for male contraception. Male hormonal contraception, which disrupts the entire spermatogenesis process, is not a preferred approach as this may lead to other long-term health issues in men.^8^

Basic reproductive research has advanced fundamental knowledge about the process and genetics of spermatogenesis. Spermatogenesis can be divided into three main phases: mitotic, meiotic, and haploid spermatid development.^9^ In the mitotic phase, spermatogonial stem cells proliferate and differentiate into differentiating spermatogonia, which subsequently enter the meiotic phase and transform into spermatocytes.^10^ Spermatocytes undergo two consecutive meiotic cell divisions to produce haploid spermatids. Spermatids then experience a multistep differentiation process and undergo dramatic morphological, molecular and cellular alterations via spermiogenesis to form spermatozoa.^11^ Although these transformations are well defined at the morphological level in most species including humans, the underlying mechanisms that regulate these intricate processes are largely unknown. Disruptions in either the mitotic or the meiotic phase tend to cause azoospermia or severe oligospermia, whereas spermiogenic defects often lead to reduced sperm counts, aberrant sperm motility and deformed spermatozoa.^11^

Knockout mouse models and *N*-ethyl-*N*-nitrosourea mutagenesis presenting with an infertile phenotype, such as spermiogenesis defects, are powerful tools with which to characterize new players in spermatid development, to determine the causes of idiopathic infertility and to develop novel therapeutic approaches for male infertility.^12, 13^ Most of the spermiogenic genes are highly conserved between mice and humans. If specific inhibitors for sperm functions can be developed for human spermatids, they would represent a new class of contraceptives that would not require disruption of early spermatogenic cell types and the testicular microenvironment. This gene-based therapies directed against the underlying cause and mechanisms of male infertility would also specifically target men with known disorders of spermatogenesis. In conjunction with improved ICSI/IVF, this novel method will offer the greatest hope for male infertility therapy.

Here we describe the currently known spermatid-unique genes involved in each of the major steps of spermiogenesis and summarize their functions in knockout mouse models. We will also discuss the mechanism, specificity, reversibility and shortcomings of emerging contraceptive ‘pill'.

## Globozoospermia-related proteins: roles in acrosome

**Figure 1:**
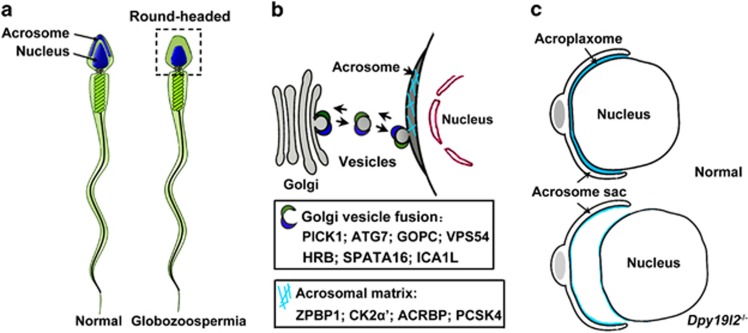
Model illustrating the role of some typical globozoospermia-related proteins in acrosome formation. (**a**) Globozoospermia is characterized by round-headed spermatozoa that lack an acrosome. (**b**) PICK1, ATG7, GOPC, VPS54, HRB, SPATA16 and ICA1L facilitate formation of trafficking vesicles from the Golgi apparatus to the acrosome. ZPBP1, CK2*α*', ACRBP and PCSK4 are proteins of the acrosomal matrix. (**c**) The absence of *Dpy19l2* leads to the destabilization of the junction between the acroplaxome and the nuclear envelope

Although the acrosome is known to be derived from the Golgi apparatus and its biogenesis involves three consecutive phases, the molecular mechanisms underlying acrosome formation remain largely unknown.^14, 15^ Globozoospermia (a condition that causes ∼0.1% of cases of human infertility) is characterized by round-headed spermatozoa that lack an acrosome (Figure 1a), and human mutants and mouse strains presenting with such a defect represent very valuable models to decipher acrosome biogenesis.^16, 17^

Using knockout mouse (−/−) models, a number of genes (e.g., *Dpy19l2*,^18^ *Pick1*,^19^ *Dnah1*,^20^ *Gopc*,^21^ *Vps54*,^22^ *Hrb*, ^23^ *Zpbp1*, ^24^ *Ck2α′*,^25^ *Hsp90b1*,^26^ *Gba2*,^27^ *Spaca1*,^28^ *Atg7*,^29^ *Smap2*,^30^ *Fads2*,^31^ *Flotillin-2*,^32^ *Ccdc136*,^33^ *Pcsk4*^34^ and *Hiat1*^35^) have been found to trigger globozoospermia. Among them, deletions and/or mutations in *SPATA16*,^36, 37^ *PICK1*,^38^ *DPY19L2*,^39^ *ZPBP1*^40^ and *DNAH1*^41^ have been identified in globozoospermia patients. The involvement of the human orthologues of the other above-mentioned mouse genes in human globozoospermia requires further investigation. It may also be important to classify these globozoospermia-related proteins in terms of their diverse cellular functions (such as Golgi vesicle fusion, acrosome exocytosis, acrosome attachment and spreading over the nucleus) or phases (Golgi phase, cap phase, acrosome phase and maturation phase). Notably, PICK1, GOPC, VPS54, HRB and SPATA16 control the Golgi vesicle fusion that is necessary for acrosome formation (Figure 1b), whereas DPY19L2 regulates the attachment of the nuclear envelope to the acroplaxome^18^ (Figure 1c). The acrosomal matrix protein ACRBP was recently identified as another globozoospermia-related protein to regulate acrosomal granule formation.^42^

It is cautious to that only a limited number of studies focus on the interaction and regulation among these globozoospermia-related proteins. *Pcsk4*-null sperm exhibit low expression of ACRBP.^34^ ATG7 regulates GOPC during acrosome biogenesis.^29^ The levels of ZPBP1 and SPACA1 are extermely low in the *Gopc*^−/−^ mouse testes.^28^ PICK1 interacts and cooperates with GOPC and CK2*α*' in acrosome biogenesis.^19^ Recently, He *et al.*^43^ identified ICA1L as a new BAR domain-binding partner of PICK1, and sperm from *Ica1l*^−/−^ mice exhibit the characteristics of globozoospermia.

In addition to acrosome integrity, acrosomal exocytosis (also called the ‘acrosome reaction') is an important event during the final phase of fertilization.^44, 45^ We emphasize the fact that most of the identified factors also control the process of exocytosis in the somatic cells of non-reproductive tissues. In particular, AFAF (also called MN9)^46, 47^ and SPESP1^48, 49, 50^ are specifically localized in the equatorial segment of sperm. We found that *Afaf*^−/−^ male mice are subfertile and the fertilization and induced acrosome exocytosis rates of *Afaf*-null sperm are considerably reduced.^51, 52^ Furthermore, AFAF facilitates an interaction with Syntaxin1a and Snap25 during sperm acrosomal exocytosis.^51, 52^ *Spesp1*^−/−^ male mice are subfertile, partially owing to the loss of equatorial membrane after the acrosome reaction. The disruption of *Spesp1* causes aberrant expression and distribution of acrosomal protein MC101, ADAM family proteins and MN9 antigen.^48^ Loss of *Afaf* or *Spesp1* does not lead to complete infertility in mice, and no mutation of either of these genes is found in male infertility patients. As such, identification of spermatid-specific factors that are critical for acrosomal exocytosis is of great interest.

## Manchette of elongating spermatids

**Figure 2:**
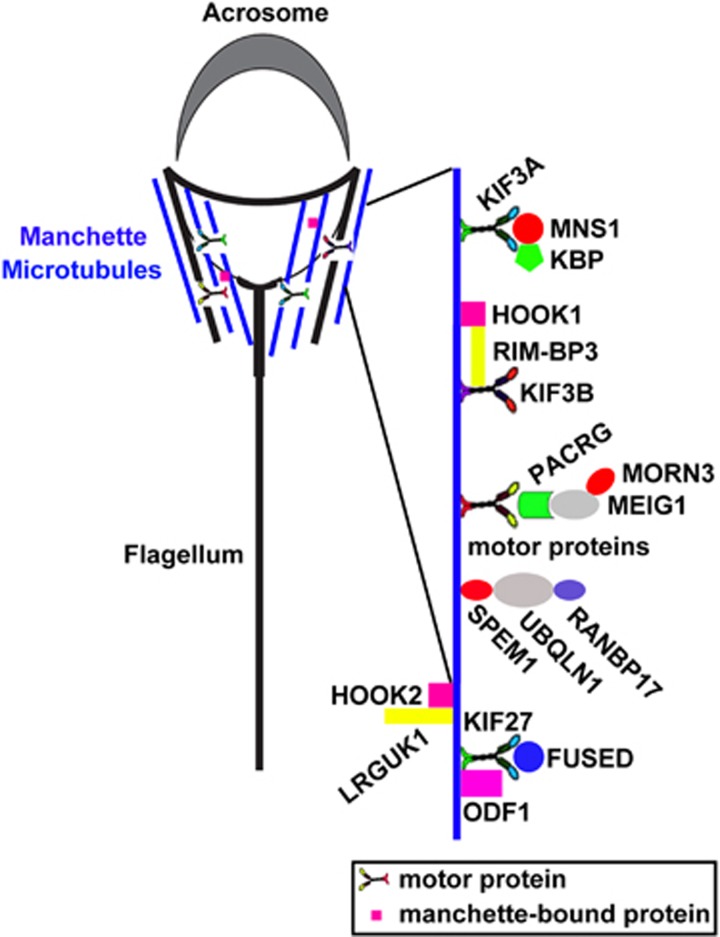
Model illustrating three typical protein complexes that leech on to manchette microtubules. The manchette is a transient skirt-like structure surrounding the elongating spermatid head and is only present during spermatid elongation. In the KIF3A/MNS1/KBP complex, MNS1 co-localizes and interacts with the KIF3A motor protein and KBP in the manchette. In the HOOK1/RIM-BP3/KIF3B complex, the RIM-BP3 protein physically associates with the manchette-bound protein HOOK1 and KIF3B motor protein. In the MORN3/MEIG1/PACRG/motor complex, the MEIG1 and PACRG form a complex and PACRG then associates with the manchette microtubules through motor protein(s). MEIG1 also interacts with the manchette-expressed protein MORN3. In the SPEM1/UBQLN1/RANBP17 complex, the SPEM1 interacts with UBQLN1 and RANBP17 in the manchette of elongating spermatids. LRGUK1 binds to HOOK2 to regulate manchette function. In the KIF27/FUSED/ODF1 complex, the FUSED interacts with the outer dense fibre protein ODF1 and manchette-expressed kinesin KIF27

The manchette is a transient skirt-like structure in the elongating spermatid head that assembles concurrently with the elongation and condensation of the spermatid nucleus and growth of the centrosome-derived axoneme.^53^ The basic platform of the manchette consists of microtubules, actin filaments and the associated motor protein (e.g., myosin).^54^ Next, we introduced the several manchette-specific protein complexes and the consequences of their disruption in spermatid elongation (Figure 2).

*Mns1*-deficient males are sterile, exhibiting a sharp reduction in sperm production, and the remnant sperm are immotile with abnormal short and crooked tails.^55^ A subsequent study has shown that MNS1 colocalizes with the motor protein KIF3A in the manchette and the principal piece of the sperm tail. Phenocopying *Mns1*^−/−^, a germ cell-specific depletion of *Kif3a*, affects sperm tail formation, manchette organization and the shaping of sperm heads.^56^ KIF3A also interacts with a KIF1-binding protein (KBP) in the manchette of elongating spermatids^57^; however, the role of KBP in spermatid elongation remains unknown.

*Rimbp3* (the gene encoding RIM-BP3) mutant mice display ectopic positioning of the manchette within the spermatid, a presumed cause of sperm head deformities.^58^ Consistent with its role in morphogenesis, the RIM-BP3 protein physically associates with HOOK1, a known manchette-bound protein required for sperm morphogenesis.^58, 59, 60^ RIM-BP3 may modulate the interaction of HOOK1 with certain organelles to which the manchette should be anchored. KIF3B, a kinesin family member, was identified as another RIM-BP3-interacting partner in a yeast two-hybrid screen.^58^

*Meig1* mutant male mice are sterile as a result of disrupted manchette structure and impaired sperm elongation and condensation.^61^ PACRG interacts with MEIG1, and *Pacrg* knockout also impairs manchette structure.^61^ MORN3, expressed in the manchette of the elongating spermatid, was recently identified as another MEIG1-interacting protein.^62^

LRGUK1 binds to HOOK2 and mutation of *Lrguk1* leads to manchette dysfunction and, ultimately, to abnormal sperm head shaping and sterility.^63, 64^ *Sun4*-deficient mice lack the linkers between the nuclear envelope and the microtubule manchette.^65, 66^ *Azi1*-null spermatids show defective manchette structure and abnormal head morphologies, suggesting defects in intramanchette transport.^67^ Inactivation of *Spem1* in mice results in deformed spermatozoa characterized by ‘head-bent-back' abnormalities and male infertility,^68^ and SPEM1 interacts with UBQLN1^69^ and RANBP17^70^ in the manchette of elongating spermatids. FUSED interacts with the outer dense fibre protein ODF1 and manchette-expressed kinesin KIF27, and *Fused*-null spermatozoa exhibit perturbed manchette formation.^71^

It will be interesting to specifically disrupt the interaction of the MNS1-KIF3A-KBP, KIF3B-RIM-BP3-HOOK1, PACRG-MEIG1-MORN3, LRGUK1-HOOK2 and UBQLN1-SPEM1-RANBP17 complex and elucidate the physiological roles of the ‘complex' in the manchette and in spermiogenesis. More mouse models affecting manchette formation can be found in other articles^72, 73, 74, 75, 76^ and review.^54^

## Head–tail conjunction: interaction between OAZ3, ODF1, SPATA6, and myosins

**Figure 3:**
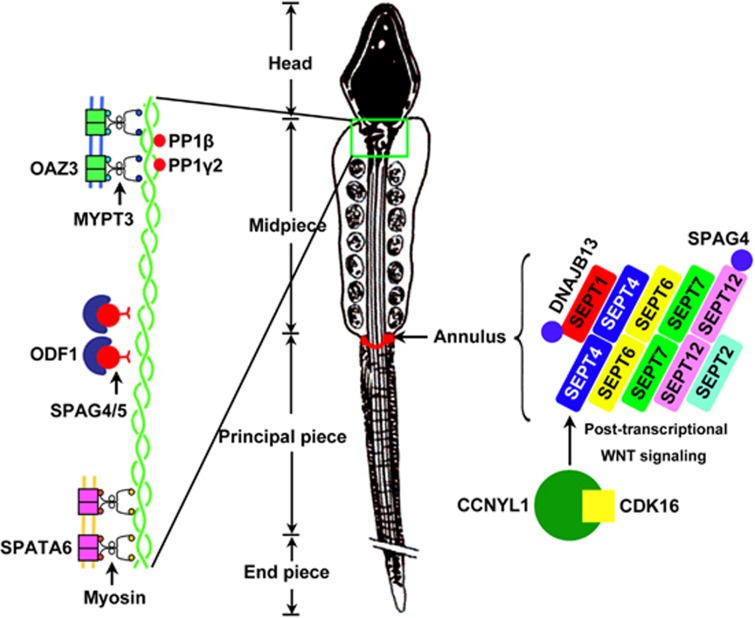
Schematic diagram of the myosin-based connecting piece and the septin-based annulus. The sperm head and tail are bridged by the connecting piece (green box), while the annulus (red structure) connects the midpiece and the principal piece of the mammalian sperm flagellum. Through interaction with myosin subunits, many factors (e.g., OAZ3, ODF1 and SPATA6) participate in the myosin-based microfilament formation and/or maintenance, which is responsible for formation of the connecting piece. *Oaz3*-encoded protein p12 interacts with MYPT3 to modulate the activity of protein phosphatase PP1*β* and PP1*γ*2. Linking of ODF1 to microtubules might occur via ODF1/SPAG5/SPAG4 interaction. SPATA6 forms a complex with myosin light and heavy chain subunits (e.g., MYL6) during connecting piece formation. The annulus is a complex of septins (SEPT) 1, 2, 4, 6, 7 and 12. These septin complexes are associated with the cochaperone DNAJB13 and SPAG4 specifically interacts with SEPT12. The CCNYL1/CDK16 complex also determines the structure and function of the annulus through posttranscriptional WNT signalling and GSK-mediated SEPT4 clustering in the epididymis

The sperm head and tail are bridged by the connecting piece, which not only serves as a physical linkage but also participates in sperm motility.^77, 78^ ‘Decapitated sperm', or ‘acephalic sperm', a type of human teratozoospermia, refers to the condition in which the ejaculate contains mostly sperm tails without heads.^79, 80, 81^ To date, several proteins that interact with myosin (an actin-based motor protein) have been reported to contribute to the connection between the sperm head and tail (Figure 3; left).

*Oaz3* encodes ornithine decarboxylase antizyme 3 and is specifically expressed in spermatids.^82^ The heads and tails of *Oaz3*-disrupted spermatids are easily separated in culture medium during incubation.^83^ Although the tailless sperm failed to acrosome-react, the heads were capable of fertilizing eggs via ICSI. Ruan *et al.*^84^ further suggest that the *Oaz3*-encoded protein p12 interacts with myosin phosphatase targeting subunit 3 (MYPT3) to modulate the activity of protein phosphatase PP1*β* and PP1*γ*2.

Similarly, mice lacking *Odf1*, a gene encoding outer dense fibre protein 1, display detachment of the sperm head.^85^ Haplo-deficiency of ODF1 (*Odf1*^+/-^) in mouse sperm causes relaxation of head-to-tail linkage and severe male subfertility.^86^ Linking of ODF1 to microtubules might occur via ODF1/SPAG5/SPAG4 (axoneme-binding proteins) interaction and to mitochondria via ODF1/KLC3 (kinesin light chain) interaction.^85^

However, neither the *Oaz3*- nor *Odf1*-deficient mice display uniformly 100% acephalic spermatozoa, suggesting that, in the absence of these genes, the connecting piece can still be formed, although in many spermatozoa it is not strong enough to maintain stability. Ablation of *Spata6* (spermatogenesis-associated 6 gene) completely disrupts formation of the connecting piece, leading to acephalic spermatozoa and male sterility in mice.^87, 88^ Interaction between SPATA6 and myosin light and heavy chain subunits (e.g., MYL6) strongly suggests that SPATA6 is involved in myosin-based microfilament transport during connecting piece formation.^88^ Further identification of spermatid-specific factors that control the connection of the sperm head and tail and mutation screening in human teratozoospermia patients remain to be investigated.

## Septin-based organization of the annulus

The annulus is an electron-dense ring structure connecting the midpiece and the principal piece of the mammalian sperm flagellum.^89^ Septin-based organization of the annulus is a requisite for the structural and mechanical integrity of the annulus. The spermatozoa from a subset of human patients with asthenospermia syndrome have a commonly disorganized annulus/septin ring.^90, 91, 92^

The genetic loss of *Sept4* in mice causes disorganization of the annulus and adjacent cortex, which results in fragility and immotility of spermatozoa.^90, 93^ Septins 1, 6, 7 and 12 co-localize with SEPT4, while SEPT12 forms a filamentous structure with septins 2, 4, 6 and 7 at the sperm annulus^89, 94^ (Figure 3; right). These septin complexes appear to assemble in round spermatids and are associated with the cochaperone DNAJB13.^95^ Recently, Yeh *et al.*^96^ determined that SEPT12 colocalizes and interacts with SPAG4 (also known as SUN4) in the nuclear periphery of round spermatids and in the tail of elongating spermatids. Furthermore, *Tssk4*^−/−^ mice are subfertile owing to disorganization of the midpiece–principal piece junction and significantly decreased sperm motility.^97, 98^ TSSK4 and ODF2 can regulate each other through phosphorylation.^98^ In addition, *Sepp1*^−/−^ and *Tat1*^−/−^ spermatozoa had structural defects similar to those described in *Sept4*-null sperm, including thinning of the flagellum at the midpiece–principal piece junction and hairpin-like bending of the flagellum.^99, 100, 101^ However, the underlying mechanism of disorganization of the annulus in *Sepp1*-null and *Tat1*-null spermatozoa and their interaction with septins remain unknown.

Cyclins comprise a family of highly conserved proteins and exert their crucial roles by activating cyclin-dependent kinases (CDKs). Close cooperation between specific sets of cyclin/CDK partners is of great significance.^102, 103^ *Cdk16*-deficient spermatozoa display thinning and elongation of the annulus region and show impaired motility.^104^ Mikolcevic *et al.*^104^ suggest that CDK16 interacts with the CCNY protein; however, *Ccny*^−/−^ male mice are fertile.^105^ CCNYL1, which is specifically expressed in the testis, also cooperates with CDK16 at the plasma membrane of sperm.^105^ In contrast to *Ccny*-null spermatozoa, spermatozoa obtained from *Ccnyl1*^−/−^ mice display significantly impaired motility and represent a thinned annulus region.^105, 106^ Accordingly, CCNYL1, but not CCNY, cooperates with CDK16 to regulate the structure and function of the annulus (Figure 3; right). The CCNYL1/CDK16 complex regulates annulus development partially via non-classical WNT (wingless-related MMTV integration site) signalling and GSK-mediated SEPT4 clustering in the epididymis.^106^

## Endoplasmic reticulum (ER) quality-control system: CALR3/PDILT/ADAMs/PMIS2

Membrane and secretory proteins are cotranslationally translocated into the ER lumen, where numerous molecular chaperones and folding enzymes assist their maturation.^107, 108^ The testes presents a special case for the control of ER protein folding because of the unusual environment (e.g., low temperature and dramatic morphological changes of spermatids).

**Figure 4:**
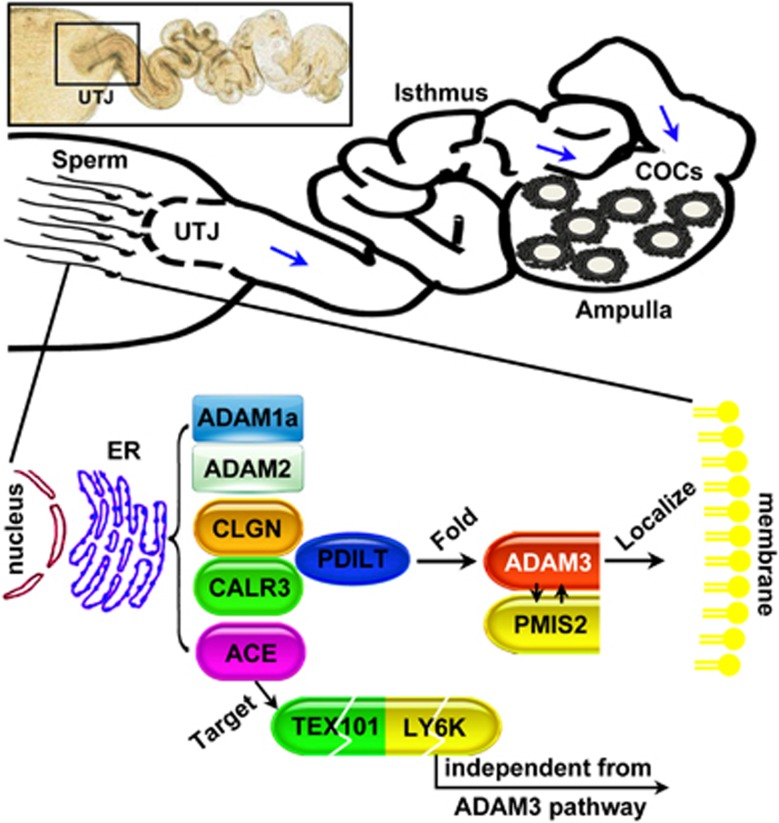
Models for the contributors and roles of the spermatid-specific ER quality-control system. After sperm are deposited in the female reproductive environment, they become metabolically active and pass through the uterotubal junction (UTJ) into the oviduct. ADAM1a, ADAM2, ADAM3, CLGN, CALR3, ACE, PDILT and PMIS2 are spermatid-specific ER chaperones. Disruption of these genes results in similar phenotypes, including impaired migration through the UTJ and/or impaired sperm-ZP binding. Heterodimerization of ADAMs and correct localization of ADAM3 are the central elements for sperm migrating and fertilizing ability. ADAM3 can regulate the PMIS2 expression directly or indirectly. ACE contributes to the removal of TEX101 and LY6K from mature spermatozoa to guarantee correct localization of ADAM3 on the mature sperm surface. Only knockout of *Ly6k* does not affect the expression and distribution of ADAM3

Calmegin (CLGN),^109^ angiotensin-converting enzyme,^110^ a disintegrin and metallopeptidase 1a (ADAM1a),^111^ ADAM2,^112^ ADAM3^111, 113^ and calreticulin 3 (CALR3)^114^ are spermatid-specific ER chaperones essential for oviduct migration and sperm–zona pellucida (ZP) binding, as disruption of these genes results in a similar sperm phenotype, that is, impaired migration into the oviduct and ZP-binding ability (Figure 4). Notably, *Adam3*-null spermatozoa can effectively fertilize eggs when surrounded in cumulus oophorus, suggesting that the principle role of ADAM3 is sperm migration into the oviduct but not ZP binding.^115^

*Prss37* deficiency causes the absence of mature ADAM3 in sperm and a defect in sperm migration from the uterus into the oviduct.^116^ *Pdilt*^−/−^ male mice are sterile because ADAM3 could not be folded properly and transported to the sperm surface without the PDILT/CALR3 complex.^115, 117, 118^ Because ADAM3 is the only protein commonly disrupted or displaced in all of the above-mentioned gene knockout sperm, it is likely that ADAM3 has a central role in sperm migration from the uterus into the oviduct. A recent study suggests that *Pmis2*-deficient spermatozoa lack the ADAM3 protein, but the amount of PMIS2 is also severely reduced in *Adam3*-deficient spermatozoa.^119^ Thus the spermatid-specific protein PMIS2 may also function as another ultimately essential factor for sperm migration and/or sperm–ZP binding. Interestingly, in contrast to previously known gene knockout mouse lines, *Ly6k*-null spermatozoa had no aberrant expression and distribution of ADAM3.^120, 121, 122^ On the other hand, LY6K is present in *Clgn*, *Calr3* and *Pdilt* mutant mouse lines, suggesting that LY6K may be a novel, ultimately essential, factor independent from the ADAM3 pathway for sperm migrating and/or fertilizing ability.

## Spermatid-specific Na^+^/H^+^ exchangers (NHEs): spermNHE and NHA1

NHEs are a family of integral membrane proteins that mediate electroneutral exchange of Na^+^ for H^+^ across the plasma membrane and have important roles in intracellular pH (pHi) regulation.^123, 124^ The SLC9 gene family encodes NHEs and can be divided into three subgroups (reviewed in Martins *et al.*^125^). Among numerous NHEs, NHE1,^126^ NHE5,^127^ NHE8,^128^ spermNHE^129^ and NHA1 (previously known as mtsNHE)^130^ are predominantly expressed in the testes. However, normal sperm motility is maintained in *Nhe1*^−/−^ mice,^131^ suggesting that the *Nhe1* gene is independent of male fertility. The role of *Nhe5* in sperm motility has yet to be determined. Surprisingly, NHE8 is highly expressed in Leydig cells, and male mice lacking the *Nhe8* gene are infertile through its effect on sterol synthesis.^128^

**Figure 5:**
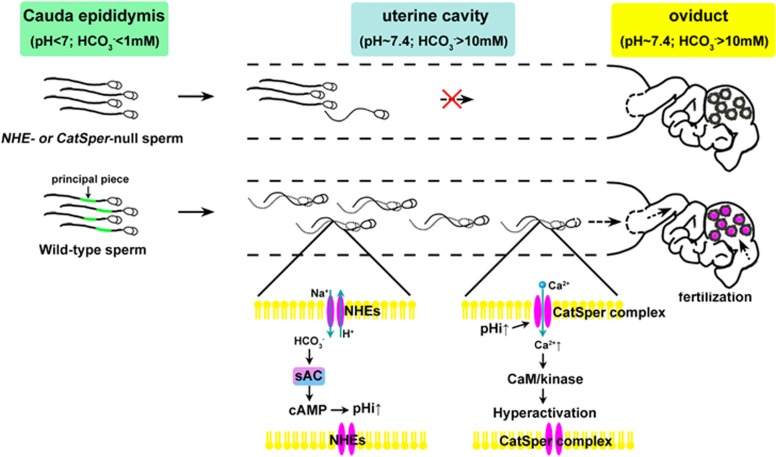
Schematic figure summarizing the critical roles of sperm-specific NHEs and CatSper. Upon ejaculation, sperm stored in the male reproductive tract enter the uterine cavity where they experience a natural decrease of pH and HCO_3_^−^. Normally, sperm adapt quickly to the environment of the female reproductive tract, particularly via Na^+^/H^+^ exchangers that are localized to the principal piece of of the sperm flagellum. However, in *spermNHE*-null and *Nha1/2*-null mice, sperm show attenuated sAC-cAMP signalling and reduced motility, resulting in failure of movement towards the ampulla of the uterine tubes to fertilize eggs. The increase in pHi activates a sperm-specific Ca^2+^ permeable channel complex known as CatSper, which allows an intracellular Ca^2+^ increase and activation of the Ca^2+^/CaM/kinase signalling pathway essential for hyperactivation

The spermNHE^129^ and NHA1^130^ are specifically distributed in the principal piece of the sperm flagellum, and both *spermNHE*- and *Nha1*-deficient male mice are completely infertile owing to the severely diminished sperm motility in the female reproductive tracts^129, 132^ (Figure 5). The sAC-cAMP signalling is impaired in spermatozoa lacking *spermNHE* or *Nha1*, and the sperm motility defect can be rescued by the addition of cell-permeable cAMP analogues.^132, 133^ The sAC is the major source of cAMP in the sperm, and male mice deficient in sAC are infertile because their sperm show no motility despite normal sperm morphology and counts.^134^

We suggest that immunization of female mice with the *Nha1*/*Nha2* (these two *Nha* genes are functionally redundant) DNA vaccine via oral feeding significantly decreases fertility rate and newborn numbers.^132, 135^ The antiserum or vaginal fluid from the *Nha1*/*Nha2* cDNA vaccinated female mice specifically recognized the principal piece of the sperm tail and triggered sperm agglutination.^132, 135^ Importantly, principal piece distribution of NHA1 in spermatozoa is phylogenetically conserved in spermatogenesis.^132^ Furthermore, the defect of the *Nha1*-null sperm is clinically relevant because NHA1 expression is either reduced or absent in patients with teratozoospermia.^136^

## Ca^2+^/CaM/kinase in the sperm

The vigorous asymmetric motion of hyperactivated spermatozoa requires Ca^2+^ entry into the sperm tail by cation channel of sperm (CatSper), a sperm-specific ion channel. CatSper is directly activated by progesterone and prostaglandins – female factors that stimulate Ca^2+^ influx.^137^ Other factors, including neurotransmitters, chemokines and odorants, also affect sperm function by changing intracellular Ca^2+^-selective current (*I*_CatSper_).^138^

CatSper1–CatSper4 are highly specialized flagellar proteins, and genetic disruption of any of the four sperm-specific CatSpers abrogated *I*_CatSper_, sperm cell hyperactivated motility and male fertility via disrupting Ca^2+^ influx^139, 140, 141, 142^ (Figure 5). Mutations in *CatSper1* and *CatSper2* are also associated with male infertility in humans^143, 144, 145^; however, *CatSper3* and *CatSper4* have not been investigated. Each of the *CatSper1–CatSper4* genes encodes a subunit of a tetramer surrounding a Ca^2+^-selective pore-forming *α* subunit. In addition to the pore-forming proteins, the sperm Ca^2+^ channel contains the auxiliary subunits CatSper*β*,^146^ CatSper*γ*^147^ and CatSper*δ*.^148^ Mice lacking the sperm tail-specific CatSperδ are infertile, and their spermatozoa lack both Ca^2+^ current and hyperactivated motility.^148^ However, the roles of CatSper*β* and CatSper*γ* and their interactions with other subunits are still unknown.

Downstream of Ca^2+^, flagellar bending is governed by Ca^2+^-binding proteins, including CaM, enkurin and calaxin.^149^ *Cris*^−/−^ male mice are subfertile owing to altered Ca^2+^ regulation of flagellar beat asymmetry.^150^ CRIS can directly interact with CaM-activated protein kinase CaMKIV and proteins involved in flagellar transport (e.g., KIF2A, IFT172).^150^ *Cnnm4*-deficient male mice are almost infertile because of perturbed Ca^2+^ influx.^151^

In addition to sperm-specific Na^+^/H^+^ exhangers and pHi-dependent CatSper (reviewed here), there are voltage-gated H^+^ channels, HCO_3_^−^ transporters and K^+^ channels, which are required for sperm pHi regulation and sperm motility (refer to Nishigaki *et al.*^152^).

## Sperm-specific glycolytic enzyme in adenosine triphosphate (ATP) production

Glycolysis is the primary source of ATP in sperm.^11^ Targeted disruption of sperm-specific glycolytic enzyme genes, such as *Gapdhs*,^153^ *Ldhc*,^154^ *Pgk2*^155^ and *Eno4*,^156^ results in reduced levels of ATP in sperm and disruption of sperm motility, leading to male infertility. Recent studies further suggest that *Galntl5*-deficient mice show male infertility owing to attenuated glycolytic enzymes (e.g., PGK2) required for motility and a patient diagnosed with asthenozoospermia had a mutation in the *GALNTL5* gene.^157, 158, 159^

## Transcriptional regulators of the haploid phase

Inactivation of *Zmynd15* in mice results in early activation of transcription of several haploid genes including *Prm1*, *Tnp1*, *Spem1* and *Catpser3*, as well as depletion of late spermatids and male infertility.^160^ The p.K507Sfs*3 mutation in exon 9 of *ZMYND15* was recently identified in one family with idiopathic azoospermia.^161^ *Rfx2*^−/−^ mice show complete male sterility owing to a complete block in development just prior to spermatid elongation. Many genes (e.g., genes required for flagellum formation and vesicle transport) are directly controlled by RFX2 during spermiogenesis.^162, 163^

## Perspectives on potential new small-molecule contraceptives targeting spermatogenic-specific proteins

**Figure 6:**
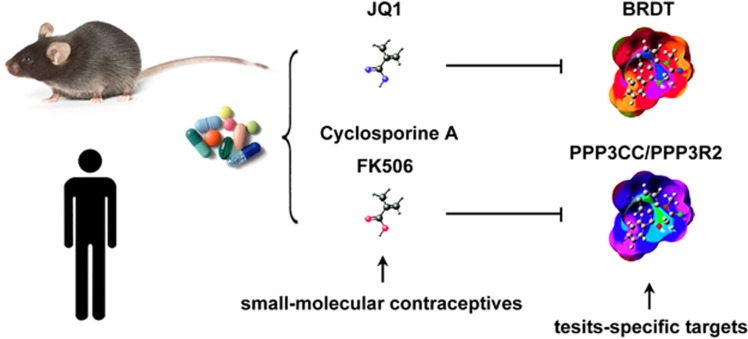
Models for the small-molecule male contraceptives. BRDT is a testis-specific contraceptive target, and JQ1 is a BRDT inhibitor that causes a reversible contraceptive effect in male mice. Sperm-specific calcineurin contains a catalytic subunit (PPP3CC) and a regulatory subunit (PPP3R2). Treatment of mice with calcineurin inhibitors, such as cyclosporine A and FK506, leads to rapid and reversible male infertility. However, side effects need to be taken into consideration

For the development of non-hormonal male contraceptives, we found that adjudin, an analogue of an anticancer drug called lonidamine, acts by disrupting germ cell-anchoring junctions in the seminiferous epithelium to induce germ cell loss from the testis.^164^ Furthermore, epidydymal protease inhibitor DNA vaccine^165^ and anti-Juno monoclonal antibody^166^ have been developed and tested for male contraception. Recently, spermatogenic-specific proteins (e.g., BRDT, PPP3CC/PPP3R2) have been pursued as potential targets of small-molecule contraceptives^167, 168^ (Figure 6).

Bromodomain, testis-specific (BRDT) is a tissue-restricted, chromatin-associated protein expressed in spermatocytes and round spermatids.^169^ BRDT is essential for chromatin remodelling during spermatogenesis and *Brdt*^−/−^ male mice are sterile, producing fewer and morphologically abnormal elongating sperm.^170^ This information provides a compelling rationale to target BRDT for contraceptive development. Filippakopoulos *et al.*^171^ have established the feasibility of targeting human BRDT with acetyl-lysine competitive small molecules (JQ1), which blocks the interactions of bromo and extra terminal (BET) proteins (BRD2, BRD3, BRD4 and BRDT) with histones. Subsequently, the complete and reversible contraceptive effect of JQ1 has been tested in mice. JQ1 treatment reduces testis size, spermatozoa number and motility without affecting hormone levels, including the serum levels of follicle-stimulating hormone, luteinizing hormone and testosterone.^167^ Although JQ1-treated males mate normally, the inhibitory effects of JQ1 evident at the spermatocyte and round spermatid stages cause a complete and reversible contraceptive effect. After JQ1 treatment is halted, the fertility of AQ-treated male mice can be completely returned.^167^

Preliminary studies confirmed that BRDT would meet target specificity, drugability and essential function requirements. However, the clinical and non-clinical safety profile of JQ1 remains to be examined. A recent study argued that mice dosed with JQ1 at efficacious doses exhibit dose-dependent decreases in their lymphoid and immune cell compartments,^172^ illustrating potential issues of immune system interference by JQ1. The development of highly selective small molecules to target BRDT specifically will avoid potential side effects owing to inhibition of its somatic isoforms (e.g., BRD2, BRD3 and BRD4). It is rather remarkable that crystal structure-based virtual screening has recently been used to identify novel potent BRDT inhibitors.^173^

Genetic disruption of either the catalytic subunit (PPP3CC) or the regulatory subunit (PPP3R2) of sperm-specific calcineurin or short-term *in vivo* pharmacological inhibition with calcineurin inhibitors (cyclosporine A or FK506) leads to complete male infertility, with reduced sperm motility owing to an inflexible midpiece during sperm maturation in the epididymis.^168^ Importantly, inhibitors of sperm-specific calcineurin could act on male fertility both effectively and reversibly because inhibition of PPP3CC/PPP3R2 targets spermatozoa in the epididymis. However, several important considerations remain. Although PPP3CC and PPP3R2 are relatively more abundant in the testis, they are still expressed in other tissues. Furthermore, cyclosporine A or FK506, used as immunosuppressant drugs, can also target the calcineurins present in other cell types. Thus an effective and safe small-molecule inhibitor will need to target only testis-specific calcineurins.^174^

We suggest that targeting spermatid-specific proteins is better, because targeting spermatids is safer and reversibility is less of an issue than targeting spermatocyte- and/or spermatogonia-specific proteins. Many spermatid-specific proteins mentioned in this review (e.g., spermNHE, NHA1, CatSper and GAPDHS) are prime candidates as targets for male contraceptive development using small-molecular inhibitors.

## Conclusions and future perspectives

Studies using mouse knockout technology have identified many spermatid-specific genes essential for spermiogenesis, which in turn are pathogenic factors of human male infertility. The large-scale analysis of mouse models will hopefully help to identify more infertility-related mutations and risk factors in humans. Importantly, the underlying mechanisms and direct correlations between spermiogenic defects of mice and male infertility in humans are far from clear. Rather than investigating novel infertility-associated factors, studies of the classification and inter-relation among known spermatid-specific factors are likely to yield the much needed information.

It is now generally accepted that sperm count and sperm morphplogy/shape are not perfect or reliable indicators of fertility and more assays (e.g., DNA fragmentation analysis, computer-assisted semen analysis, motile sperm organelle morphology examination^175^ and gene mutation screening) need to be adapted for clinical use.^176^ Some obvious structural defects can be detected microscopically, whereas some defects in energy production, signalling transduction and metabolism require more sophisticated assays for screening. Although the molecular diagnosis of infertility would be difficult with the current available technologies, we suggest that deletions and/or mutations in *SPATA16*,^36, 37^ *PICK1*,^38^ *DPY19L2*,^39^ *ZPBP1*,^40^ *DNAH1*,^41^ *CatSper1*/*2*,^143, 144, 145^ *GALNTL5*^158^ and others^7^ need to be checked for infertility diagnosis of globozoospermia. Whole genome-based techniques will hopefully help to identify more infertility-related mutations and risk factors in future.

A male contraceptive ‘pill' is still many years away. But recent research into developing small-molecule inhibitors that target specific sperm antigens/enzymes/proteins as contraceptives may offer some insightful information in male contraceptive development.

## Abbreviations

ART: assisted reproductive techniques
IVF: *in vitro* fertilization
ICSI: intracytoplasmic sperm injection
CDK: cyclin-dependent kinase
ER: endoplasmic reticulum
ADAM: a disintegrin and metallopeptidase
ZP: sperm-zona pellucida
NHE: Na^+^/H^+^ exchanger
pHi: intracellular pH
CatSper: cation channel of sperm
*I*_CatSper_: intracellular Ca^2+^-selective current
ATP: adenosine triphosphate
BRDT: bromodomain, testis-specific
